# Trace Elements in Portuguese Children: Urinary Levels and Exposure Predictors

**DOI:** 10.3390/toxics11090767

**Published:** 2023-09-09

**Authors:** Luísa Correia-Sá, Virgínia C. Fernandes, Maria Luz Maia, Edgar Pinto, Sónia Norberto, Agostinho Almeida, Cristina Santos, Cristina Delerue-Matos, Conceição Calhau, Valentina F. Domingues

**Affiliations:** 1 REQUIMTE/LAQV, Instituto Superior de Engenharia do Porto, Instituto Politécnico do Porto, Rua Dr. António Bernardino de Almeida 431, 4249-015 Porto, Portugal; virginiacruz@graq.isep.ipp.pt (V.C.F.); mariadaluz.maia@gmail.com (M.L.M.); cmm@isep.ipp.pt (C.D.-M.); 2 Center for Research in Health Technologies and Information Systems, 4200-450 Porto, Portugal; sonia.norberto.nutri@gmail.com (S.N.); csantos.cristina@gmail.com (C.S.); ccalhau@nms.unl.pt (C.C.); 3 REQUIMTE/LAQV, Departamento de Ciências Químicas, Faculdade de Farmácia, Universidade do Porto, 4249-015 Porto, Portugal; ecp@ess.ipp.pt (E.P.); aalmeida@ff.up.pt (A.A.); 4 Departmento de Saúde Ambiental, Escola Superior de Saúde, Instituto Politécnico do Porto, R. Dr. António Bernardino de Almeida 400, 4200-072 Porto, Portugal; 5 Health Information and Decision Science, Faculty of Medicine, University of Porto, 4200-319 Porto, Portugal; 6 Nutrição e Metabolismo NOVA Medical School, Faculdade de Ciências Médicas, Universidade Nova de Lisboa, 1169-056 Lisboa, Portugal

**Keywords:** elements, biomonitoring, children, obesity, predictors, urinary levels

## Abstract

Exposure to environmental chemicals during developmental stages can result in several adverse outcomes. In this study, the exposure of Portuguese children to Cu, Co, I, Mo, Mn, Ni, As, Sb, Cd, Pb, Sn and Tl was evaluated through the analysis of first morning urine through ICP-MS. Furthermore, we attempted to determine possible exposure predictors. The study sample consisted of 54% girls and 46% boys, with a median age of 10 years; 61% were overweight/obese and were put on a nutritionally oriented diet. For I, half of the population was probably in deficiency status. The median urinary concentrations (μg/L) were Cu 21.9, Mo 54.6, Co 0.76, Mn 2.1, Ni 4.74, As 37.9, Sb 0.09, Cd 0.29, Pb 0.94, Sn 0.45, Tl 0.39 and I 125.5. The region was a significant predictor for Cu, Co, Ni, As and Tl. Children living in an urban area had higher urinary levels, except for Co and Ni. Age was a significant predictor for Cu, I, Mo, Mn, Ni, Sb, Cd and Sn with urinary levels of these elements decreasing with age. No sex-related differences were observed. Diet and weight group were predictors for urinary Cu, Mn, Ni, Sb and As. Significant differences were observed between the diet/weight groups for Cu, Ni, Sb and As, with the healthy diet group presenting higher values.

## 1. Introduction

Trace elements are naturally occurring chemical elements and their environmental presence and distribution is a consequence of both natural processes and anthropogenic activities [1,2].

According to a widely accepted classification, elements can be categorized in three groups: essential elements, probably essential elements, and potentially toxic elements [3]. Elements such as Co, Cu, Mo and I are considered essential nutrients, playing a crucial role in metabolism; they are co-factors of many enzymes and have a variety of biochemical and physiological functions in living organisms. However, at excessive levels in the human body, above specific reference levels, they become toxic to human health [3]. 

Less is known about the functions of probably essential elements, such as Mn and Ni, but it is admitted that they may have a specific beneficial action in humans [4]. Potentially toxic elements can be hazardous to human health even when present in low concentrations. Included in this category are Cd, Pb, Sb, As, Tl and Sn [3,4].

Humans are exposed to chemical elements through food, air and water. In non-occupationally exposed individuals, food is the most important source of most elements [5,6]. Children are a particularly susceptible population, with their still developing systems, hand-to-mouth behavior and playing proximity to the floor, which can lead to inadvertent ingestion of contaminants present on surfaces and a higher uptake than adults (higher food intake per body weight) [7,8,9].

While estimates of the burden of disease specifically attributable to environmental chemicals in children are limited, there is clear scientific evidence that exposure to environmental chemicals during different developmental stages can result in a number of adverse outcomes, and thus it is clear that reducing exposure could have a significant positive impact on children’s health and well-being [10,11]. In utero exposure to trace elements has been linked with an increased risk for preterm birth [12] and harmful effects on fetal development [13]. Metals such as Pb seem to be potent neurotoxins that can affect the cognitive and behavioral development of children, even at low levels of exposure [14]. Significantly decreased Pb and Cd urinary values were observed in autistic children [15].

Trace elements can act as major endocrine disrupters and therefore seem to be risk factors for impaired thyroid health and the evolution of thyroid cancer [16], immune function [17] or even metabolic syndrome and obesity [18,19,20,21,22,23,24,25,26]. 

Although human biomonitoring studies and data on trace elements are available for several populations, namely the US [27], Canada [28] and Germany [29], to our knowledge, there are no human biomonitoring studies for the Portuguese population. The scarcity of exposure assessment information on the Portuguese population prompted us to develop this study with the intention to obtain preliminary data on urinary levels of selected trace elements in children aged 4 to 18 years old, with normal and excess weight, from two Portuguese regions. Furthermore, we attempted to determine possible exposure predictors.

## 2. Materials and Methods

### 2.1. Study Subjects and Sample Collection

Participants were recruited in the pediatric appointment at Hospital de São João, Porto, and in several local schools, in 2014 and 2015. They lived in Porto and Aveiro, two districts in the north and central regions of Portugal (79 children from Porto and 31 from the Aveiro region). A total of 110 children (55 boys, 55 girls) were enrolled in the study, with an age range of 4 to 18 years (arithmetic mean ± standard deviation: 10.4 ± 3.4 years).

Two groups of children were considered. Group 1 included children diagnosed with obesity/overweight but with no other known associated diseases. Recruited from the pediatric nutrition consultation, they were on a healthy diet for at least three months, based on fresh and varied foods and a few packaged and processed foods. The children in group 2 were of normal/low weight and maintained their regular diet.

First morning urine samples were collected from each participating child. All samples were kept cool during transportation and then stored at –20 °C until analyses. The study was approved by the Ethics Committee of the Centro Hospitalar Universitário de São João/FMUP (Faculdade de Medicina da Universidade do Porto; ref. 163.13) and all parents gave their written consent.

### 2.2. Determination of Creatinine

Urinary creatinine concentration was determined by modified Jaffé’s method [30] in an Olympus AU5400^®^ Chemistry Analyzer (Beckman-Coulter^®^, Brea, CA, USA) at Hospital São João, Department of Clinical Pathology. 

### 2.3. Determination of Trace Elements

Twelve trace elements were analyzed by inductively coupled plasma mass spectrometry (ICP-MS), including essential and probably essential elements (i.e., Cu, Co, Mn, Mo, Ni and I), potentially toxic elements toxic elements (i.e., Sb, Sn, Tl, As, Cd and Pb). Analyses were performed using an iCAP™ Q instrument (Thermo Fisher Scientific, Bremen, Germany), equipped with a MicroMist™ nebulizer, a baffled cyclonic spray chamber (Peltier-cooled), a standard quartz torch, and a two-cone design (nickel sample and skimmer cones). An in-house method was developed based on the CDC Laboratory Procedure (method no. 3018.3 and 3018A.2; revised 19 March 2012) [31] for the analysis of Cu, Co, Mn, Mo, Ni, Sb, Sn, Tl, As, Cd and Pb. And for iodine urinary analysis a method based in the CDD was applied, described in our previous work [32]. A high purity argon (99.9997%) was used as the nebulizer and plasma gas. The operating parameters of the ICP-MS instrument were as follows: RF power 1550 W; plasma gas flow 14 L/min; auxiliary gas flow 0.8 L/min; nebulizer flow rate 0.95 L/min. Control of the equipment and data acquisition was performed with Qtegra software 2.14.5122.158 (Thermo Fisher Scientific, Waltham, MA, USA).

The elemental isotopes ^45^Sc, ^89^Y, ^115^In, ^159^Tb and ^103^Rh were used as internal standards. Calibration curves were obtained with seven calibration standard solutions (in the range of 10–1000 µg/L) prepared by appropriate dilution of multi-element standard stock solutions with 2% HNO_3_. The internal standard solution was added to all samples and standard solutions to obtain a final concentration of 10 µg/L.

For iodine analysis, the ^127^I isotope was monitored for analytical determination, and the ^125^Te isotope was monitored as an internal standard. The calibration curve was obtained with six solutions of iodine concentrations within the 25–1000 µg/L range. The calibration standard solutions were prepared by adequate dilution of the iodine standard (Plasma CAL, SCP Science, Baie-d’Urfé, Quebec, Canada). 

The internal standard solution was added to all samples and to the standard solutions to obtain a 10 µg/L final concentration. All the solutions were prepared with 1.0% (*v*/*v*) tetramethylammonium hydroxide, TMAH 25% *w*/*w* (Alfa Aesar, Karlsruhe, Germany), 0.01% Triton™ X-100 (Sigma-Aldrich, St. Louis, MO, USA) and 10 µg/L Te (Sigma-Aldrich, St. Louis, MO, USA).

The instrument was tuned daily for maximum sensitivity and signal stability, as well as for the lowest formation of oxides and doubly charged ions using the Thermo iCAP Q Tuning Solution (1 µg/L of Ba, Bi, Ce, Co, In, Li, and U in 2.5% HNO_3_ + 0.5% HCl; Inorganic Ventures, Christiansburg, VA, USA). For analytical quality control purposes, certified reference materials Trace Elements Urine L1 and L2 (Seronorm™, Sero, Billingstad, Norway) were analyzed under the same conditions as the samples. The results obtained are within the ranges established in the CRM materials, thus showing the validation and robustness of the analytical methodology.

Calibration curves showed good linearity, with determination coefficient values higher than 0.99 for all the analyzed elements. Trace Elements Urine L1 and L2 were reanalyzed together with a blank and an intermediate calibration standard every 20 samples. Additionally, 1 in every 20 samples was reanalyzed at the end of each session. The limits of detection (LODs) achieved ranged from 0.1 to 1.5 ng/mL. Inter-day precision (%RSD) calculated through different days was, in all cases, below 11%.

Concentrations of trace elements were expressed both as μg/L (unadjusted concentrations) and μg/g creatinine to control for differences in urine dilution.

### 2.4. Sample Preparation

Urine samples were diluted (1:10) with 2% (*v*/*v*) HNO_3_ (≥69.0%, TraceSELECT^™^, Fluka^™^, Honeywell Research Chemicals, Charlotte, NC, USA). The urine pool used to prepare the calibration standards was prepared by mixing several urine samples. Prior to ICP-MS analysis, the diluted samples were vortexed and centrifuged at 4500 rpm for 5 min. 

### 2.5. Statistical Analysis

Statistical analysis was performed using SPSS 20.0 (IBM Corporation, Armonk, NY, USA). Basic descriptive statistics (median, geometric and arithmetic means, range and percentiles) were calculated. Regarding age, the participants were divided according to European regulations for clinical studies in pediatric patients [33]: (i) children—2 to 11 years of age; (ii) adolescents—12 to 18 years of age. Mann–Whitney U tests were performed to assess possible differences between groups regarding age, sex, weight, height, BMI and urinary creatinine concentration.

Since urinary concentrations of trace elements (both creatinine-normalized and non-normalized values) had a non-normal distribution (assessed by Kolmogorov–Smirnov test), non-parametric tests were applied. Mann–Whitney U tests were performed to assess possible differences between diet/weight groups, age, gender and region for the urinary values (µg/g creatinine).

Principal component analysis (PCA) was used to concisely represent how the exposure predictors, namely age, region, gender, weight and diet group, and the urinary element levels were correlated for creatinine. The selection of factors to be used as predictors for urinary levels in regression models was guided by scree plots and eigenvalues exceeding 1.0, following the Kaiser criterion [34]. We maintained factor loadings and correlations between exposure variables and factors greater than 0.3 [34].

To evaluate the influence of different factors (e.g., diet, age, gender, region) on urinary trace element levels, a multiple log-linear regression analysis was performed using the ln-transformed concentration data (µg/g creatinine) as a dependent variable. 

The changes in urinary concentration by a unit change in the independent variables are presented as geometric mean ratios (gMR) along with their corresponding 95% confidence intervals (95% CI), which can be interpreted like odds ratios (OR). For this purpose, the regression beta coefficients were back transformed to their natural scale (exp (β)). A *p*-value ≤ 0.05 was always considered statistically significant.

## 3. Results and Discussion

### 3.1. Study Subjects

Urinary concentrations of a broad panel of trace elements were analyzed in urine samples collected from n = 110 Portuguese children, aged 4 to 18 years. The study sample consisted of 50% girls and 50% boys, with a median age of 10 years. Most of the children (61%) were overweight/obese and were put on a nutritionally oriented diet (healthy diet group; n = 67). The remaining children were normal weight (except one, who was underweight) and constituted the regular diet group (n = 43). More detailed information about the two groups is given in Table 1. Although the discriminator’s body weight and BMI naturally differed significantly between the two groups, age, sex, height, and urine creatinine were quite similar (Table 1). Four of the one hundred ten children had urinary creatinine values below 0.3 g/L. However, all urine samples were included in the statistical analysis, since creatinine concentrations below 0.3 g/L, the cutoff for adults [35], do not necessarily indicate excessive dilution in children but may simply be due to lower muscle mass [36,37].

### 3.2. Urinary Levels

The results of urinary trace elements’ levels for the entire sample of children are summarized in Table 2.

While the urinary concentrations of various elements generally remained below the biomonitoring reference values, including RV95, HBM-I and II and BE, some of the analyzed elements presented median values above those thresholds, namely Ni and As (median values of 4.74 μg/L and 37.9 μg/L, respectively). For elements such as Cu, Pb, and Tl, the 95th value was considerably higher than the reference values. For Cd, Co and Sn, our maximum values exceed the reference values, and finally, for the rest of the analyzed elements, the median and even maximum values were below the available reference data (Table 2). Most of the exceedances observed in our study refer to RV95 values; however, for Sn one child exceed the BE value. These findings shed light on the chemical prioritization for subsequent investigation and emphasize the importance of delving into the origins of these exposures. 

On the opposite, the urinary iodine values for our children were below the recommended. For iodine, only 39.1% of the children had values in the 100–199 µg/L range, which, according to the WHO, indicates an adequate iodine intake [39]. The percentage of children with urinary concentrations below 100 µg/L was 50.9%, which means that half the studied sample did not meet the WHO criteria and was probably in iodine deficiency status.

In a recent study with Portuguese school-age children, the percentage of values below 100 µg/L was significantly lower (32%). Additionally, the authors found that the risk of a urinary iodine concentration lower than 100 µg/L was significantly related to milk consumption [32].

Nonetheless, the obtained median urinary concentrations for the Portuguese children fell within the range reported for biomonitoring studies in other countries (see Table 3). Regarding essential and probably essential elements, Portuguese children showed lower urinary levels of Cu and higher urinary levels of Mn than reported for Italian and Spanish children [40,41]. The median value for Mn (1.80 μg/L) was around three to four times higher in Portuguese children than in Italian [41] and Spanish children [40], respectively. Mn is essential to many functions, such as bone mineralization, metabolic regulation and protection against oxidative processes, but in excess, it can also be a potent neurotoxin due to accumulation in the brain [42]. Mn is widespread in the environment with studies connecting human exposure with drinking water, air and even food consumption [42].

For the potentially toxic elements, (As, Cd, Ni, Pb, Sb, Sn and Tl) geometric mean values ranged from 0.08 μg/L for Sb to 33.9 μg/L for As. When comparing with Spanish, Italian and US children, similar values were obtained for Ni, Sb and Sn. However, for As, Pb, Cd and Tl in general, higher values were observed in the present study [27,40,41].

For Pb, higher levels were obtained (GM: 1.03 μg/L) compared to those reported in US children (0.20–0.26 μg/L) [27]. In human biomonitoring studies, blood Pb is the preferred biomarker to assess exposure, but urinary levels may better reflect recently absorbed Pb, although there is greater intra-individual variation in urinary Pb [27].

In a study carried out in 2014 by Reis et al. [43] in Lisbon, it was shown that significant fractions of Pb occurred in bio-accessible forms in various leisure areas, including public gardens, parks, playgrounds and schoolyards. In another study, dietary Pb intake was determined in duplicate diet samples provided by 30 participants working or studying at the University of Aveiro (Portugal), and Pb was detected in all samples analyzed, with values ranging between 0.009 and 0.10 mg/kg wet weight [44]. Reis et al. [45] evaluated the exposure of Portuguese children under 6 years, living near incinerators facilities, to heavy metals (in the blood); however, no significant differences were observed between Cd and Pb blood levels between exposed and control groups. Nonetheless, the obtained blood data were above the reference values [45].

The As median level (33.9 µg /L) was considerably higher than that obtained in American children (4.9–5.0 µg/L) [27], but a similar value was obtained in Spanish children from the Valencia region [40]. The human exposure risk to the environmental As in Portugal, in 2007, was assessed by combining drinking water levels and atmospheric deposition (assessed by tap water analysis and a moss biomonitoring survey) [46]. The authors identified the northern interior and the central region of the country, where Aveiro is located, as the regions with the highest water and atmospheric levels of As. The general population is exposed to various forms of As, with very different toxicity, from toxic inorganic species (e.g., arsenite, arsenate), which are classified as group 1 carcinogens (IARC, 2017) to non-toxic organic species (e.g., arsenobetaine, arsenocholine). The main sources of exposure to inorganic As are drinking water and food, mainly cereals and pulses, while seafood is the main source of organic species [40]. It is important to note that As is widely present in water, coming from different origins, and crops that are irrigated with large amounts of water (such as rice) typically have higher levels of As compared to other foods [47]. A recent review was conducted evaluating available human biomonitoring data for the Portuguese population where metals and metalloids were included [48]. From these studies, only one reported urinary data for the populations living near and working in the Panasqueira mine [49], and results showed levels of As similar to the ones determined for our population. The authors believe these values were related to mine contamination and that this environmental contamination probably persists after mines are closed [48,49].

The results of the present study refer to total As in urine. Urinary total As levels in about 90% of children tested were within the normal range of <100 µg/L accepted by the Agency for Toxic Substances and Disease Registry [50] However, it would be important to conduct a speciation study to verify which As species are present in the urine and thus conclude the real toxicological significance of the exposure.

The median value for Cd (0.25 μg/L) was similar to that of Italian children [41] but higher than that of Spanish [40] and American children [27]. Likewise, the urinary level of Tl was higher in Portuguese children, about three times higher than for US [27] and Spanish [40] children. Nonetheless, a maximum value of the maximum value of 2.68 μg/L (Table 2) was obtained. Humans are primarily exposed to Tl through the consumption of contaminated food or drinking water [51].

Data from the Portuguese Total Diet Sample Pilot Study, where Pb, As, Cr and Cd profiles were assessed, show the highest amount (μg/kg) obtained was: As 9138 ± 237 in octopus; Pb 282 ± 5.5 in snails; Cr 605 ± 28 in cured meat; and Cd 248 ± 5 in snails. However, according to the authors, these levels did not pose a risk to the population [52].

In the DEMOCOPHES project, Cd was evaluated in urine samples from mother–child couples from 16 European countries, including Portugal [53]. The main predictors of exposure were smoking, age, educational level, and rural residence. The geometric mean values for children differed by a factor of 7 between the lowest (in Denmark and Romania) and the highest concentration (United Kingdom and Luxembourg) at the country level [53]. Authors determined differences in Cd exposure distributions, but it was not clear if these differences were related to differences in national contamination level of the environment or foods available on the market or to differences in lifestyle or dietary patterns [53].

**Table 2:** Results of the determination of trace elements in the urine (µg/L and µg/g creatinine) of Portuguese children (n = 110; aged 4–18 years) and reference values.

This Study										Biomonitoring Guidance Value					
Elements	LOD	μg/L				μg/g Creatinine									
	[µg/L]	Min	Median	95th	Max.	Min	Median	95th	Max.	Age Group	Agency	Type	Value	Units	Ref
Cooper (Cu)	0.11	5.30	21.9	52.6	94.4	5.05	19.9	40.6	50.2	3 to 5	Canada (2009–2011)	RV95	29	µg/L	[54]
										6 to 19	Canada (2009–2011)	RV95	25	µg/L	[54]
Iodine (I)	0.89	16.39	125.5	302.6	536.9	20.65	122.52	213.8	316.3	6–10	ATSDR (2004)	BE	360	µg/L	[55]
											IPCS (2009)	BE	360	µg/L	
											IOM (2001)	BE	580	µg/L	
											ATSDR (2004)	BE	470	µg/g creatinine	
											IPCS (2009)	BE	470	µg/g creatinine	
											IOM (2001)	BE	760	µg/g creatinine	
										Adults	ATSDR (2004)	BE	450	µg/L	
											IPCS (2009)	BE	450	µg/L	
											IOM (2001)	BE	730	µg/L	
											ATSDR (2004)	BE	450	µg/g creatinine	
											IPCS (2009)	BE	450	µg/g creatinine	
											IOM (2001)	BE	730	µg/g creatinine	
Molybdenum (Mo)	0.25	8.38	54.6	112.2	149.8	10.99	51.53	101	200.6	not specified	Health Canada (2016)	BE	7516	µg/L	[56]
											US EPA (1992)	BE	206	µg/L	[57]
											RIVM (2000)	BE	442	µg/L	
											IOM (2001)	BE	1326	µg/L	
											OECD SIDS (2013)	BE	7516	µg/L	
											US EPA (1992)	BE	206	µg/g creatinine	
											RIVM (2000)	BE	442	µg/g creatinine	
											IOM (2001)	BE	1326	µg/g creatinine	
											OECD SIDS (2013)	BE	7516	µg/g creatinine	
										6–19	Health Canada (2009–2011)	RV95	230	µg/L	[54]
										3–5		RV95	290	µg/L	
Cobalt (Co)	0.0083	0.13	0.76	1.81	2.89	0.14	0.69	2.41	3.26	>17	Mayo Clinic	Clinical Interpretative value	<1.7	µg/g creatinine	[58]
Manganese (Mn)	0.0074	0.50	2.1	8.04	27.9	0.33	1.90	4.19	12.2	>18	Mayo Clinic	Clinical Interpretative value	<40	µg/g creatinine	[58]
Nickel (Ni)	0.069	1.13	4.74	12.2	24.8	1.05	4.18	8.9	35.2	3 to 14	Germany (2003–2006)	RV95	4.5	µg/L	[59]
										3 to 79	Canada (2009–2011)	RV95	4.4	µg/L	[54]
Arsenic (As)	0.27	3.95	37.9	170.3	435.7	6.19	30	158	610.4	3 to 14	Germany (2003–2006)	RV95	15	µg/L	[59]
										3 to 79	Canada (2009–2011)	RV95	27	µg/L	[54]
Antimony (Sb)	0.0023	0.02	0.09	0.28	0.32	0.02	0.08	0.16	0.20	3 to 14	Germany (2003–2006)	RV95	0.3	µg/L	[59]
										3 to 79	Canada (2009–2011)	RV95	0.17	µg/L	[54]
Cadmium (Cd)	0.0062	0.05	0.29	0.58	0.72	0.08	0.27	0.52	1.08	children and adolescents	HBM Commission (2011)	HBM-I	0.5	µg/L	[60]
										children and adolescents	HBM Commission (2011)	HBM-II	2	µg/L	
										3 to 14	Germany (2003–2006)	RV95	0.2	µg/L	[59]
										3 to 5	Health Canada (2009–2011)	RV95	0.69	µg/L	[54]
										6 to 19		RV95	0.68	µg/L	
Lead (Pb)	0.016	0.31	0.94	7.57	19.9	0.19	0.90	4.08	17.1	3 to 5		RV95	1.7	µg/L	
										6 to 19		RV95	1.3	µg/L	
Tin (Sn)	0.0052	0.08	0.45	3.8	30.1	0.06	0.40	2.81	37	not specified	Health Canada (2016)	BE	20	µg/L	[61]
										not specified		BE	26	µg/g creatinine	
Thallium (Tl)	0.0079	0.01	0.39	1.37	2.68	0.06	0.38	1.03	1.84	3 to 14	Germany (2003–2006)	RV95	0.6	µg/L	[59]
										3 to 5	Canada (2009–2011)	RV95	0.64	µg/L	[54]
										6 to 19	Canada (2009–2011)	RV95	0.59	µg/L	

ATSDR—Agency for Toxic Substances and Disease Registry; IPCS—World Health Organization (WHO) International Program on Chemical Safety; IOM—Institute of Medicine; US EPA- United States Environmental Protection Agency; RIVM—Netherlands National Institute for Public Health and the Environment; OECD SIDS—Organization for Economic Cooperation and Development Screening Information Dataset; BE—biomonitoring equivalents; RV95—Reference value 95; HBM-I—Human Biomonitoring guidance value I; HBM-II—Human Biomonitoring guidance value II.

**Table 3:** Geometric mean values (µg/L) of the studied Portuguese children versus other biomonitoring studies.

Countries	Sampling Period	Age (Years)	n	Cu	Co	Mo	Mn	Ni	As	Sb	Cd	Pb	Sn	Tl
Europe (16 European countries) [53]	2011–2012	5–12	1680	n.a.	n.a.	n.a.	n.a.	n.a.	n.a.	n.a.	0.07 *	n.a.	n.a.	n.a.
Italy [41]	2007–2008	5–11	110	37.9	0.99	n.a.	0.68	6.80	n.a.	0.07	0.38	1.24	1.25	n.a
Spain [40]	2015	6–11	120	35.3	1.43	63.2	0.43	4.32	33.9	0.79	0.18	1.18	n.a	0.19
US [27]	2015–2016	6–11	379	n.a.	0.53	56.2	<0.13	n.a.	4.89	0.06	<LOD	0.26	0.87	0.17
		12–19	402	n.a.	0.57	47.7	<0.13	n.a.	5.00	0.06	0.05	0.20	0.49	0.17
Portugal (this study)	2014–2015	4–18	110	18.6	0.65	48.1	1.80	4.20	33.9	0.08	0.25	1.03	0.48	0.36

n.a.—not analyzed; n—number of children; * value in μg/g creatinine.

### 3.3. Predictors of Exposure

Table 4 describes the PCA results. As mentioned in the section on methods and materials, only variables with eigenvalues exceeding 1.0 were considered; thus, gender was excluded. A Kaiser–Meyer–Olkin value of 0.728, and a significance level for the Bartlett’s test below 0.001 was obtained, suggesting substantial correlation in the data [34]. Five factors were retrieved that accounted for 68.4% of the variance. Factor 1 is composed by weight and diet group (obese/overweight on a diet versus normal weight/underweight following the usual diet), region, Cu, Mn, Ni, Sb, As and Pb. Factor 2 is composed by age, Cu, I, Mo, Sb, Cd and Tl. On the other hand, factor 3 is composed by weight and diet group, region, Co, Ni and Tl. In factor 4 are included Mo, Cd and Sn. And finally, factor 5 is composed by weight and diet group and As and Tl.

Analyzing the results in more detail, variables that have shown to be associated with significant differences in urinary trace element levels (Mann–Whitney tests, see Table S1 in Supplementary Materials), and tested in the PCA, were considered for multiple log-linear regression analysis (see Table 5). The calculated final models accounted for more than 10% of the variance, except for As and Co.

Geographic location (region) was a significant predictor for Cu, Co, Ni, As and Tl. Between the two regions groups, Porto and Aveiro, there were significant differences (*p*-values from <0.0001 to 0.016), with children living in the urban area of Porto showing higher values, except for Co and Ni. This is an expected finding, also observed by other authors, namely for As and Co [40], indicating a higher exposure of children living in urban areas. Children in the Aveiro region presented around 1.72 times higher urinary Ni values than Porto region children. This highlights the importance of further human and environmental monitoring studies to unveil the possible sources of exposure responsible for the differences between the two regions.

On the other hand, age is correlated with Cu, I, Mo, Mn, Ni, Sb, Cd and Sn beta values showing a negative association, i.e., the urinary levels (µg/g creatinine) of these elements decreased with age. This negative association was also observed by Rocca et al. (2016) for Mo. Previous studies have also reported similar findings, with the authors attributing this to different dietary patterns, gastrointestinal absorption and metabolism [40,41,53]. Costa-Leite al. 2017 also found higher values for urinary iodine for younger children. Furthermore, these authors concluded that iodine status was closely related to milk consumption, with older children reporting lower milk consumption [32] presenting lower urinary iodine levels.

Diet and weight group (obese/overweighted children on a healthy diet *vs* normal/underweight children on their regular diet) were predictors for urinary Cu, Mn, Ni, Sb and As, with the obese/overweight children on a healthy diet presenting higher values, except for As. A direct relationship between serum Cu levels and overweight/total obesity has been reported in children and adolescents aged 6–18 years [62]. Other authors also found that individuals with a metabolically unhealthy phenotype had higher urinary levels of Cu and Pb than those with a healthy phenotype (all *p*-values < 0.05) [63]. Regarding Sb, a recent study found a curvilinear relationship between urinary levels and obesity, with the moderate-level group having the highest odds of obesity [64]. 

Recently, Warwick, M. et al. found a strong inverse relationship between body measures and the daily excretion of urinary As metabolites [65]. In the present, our obese and overweight children also presented lower urinary As values. For Sn, a study with obese and overweight children also found higher urinary levels compared to normal weight children [66]. 

Food intake is the main route of exposure for several of the studied trace elements. Consequently, it is advisable to gather more accurate dietary data in forthcoming research to conduct a comprehensive examination of the primary food sources. Consequently, the present results highlight the need for further studies in the Portuguese population to assess possible sources of exposure and thus reduce exposure.

## 4. Conclusions

This study provides information on exposure to various trace elements in Portuguese children. Overall, the results fell within the range found in other child populations worldwide.

It was also possible to obtain valuable information on exposure predictors and differences between population subgroups. The median urinary concentrations (μg/L) were Cu 21.9, Mo 54.6, Co 0.76, Mn 2.1, Ni 4.74, As 37.9, Sb 0.09, Cd 0.29, Pb o.94, Sn 0.45, Tl 0.39 and I 125.5.

Age proved to be the most important predictor of exposure to most of the elements analyzed, with younger children presenting higher urinary values for Cu, I, Mo, Mn, Ni, Sb, Cd and Sn.

The geographic location (region) was a significant predictor for Cu, Co, Ni, As and Tl. Children living in an urban area (Oporto City) had a higher level, except for Co and Ni. Age, on the other hand, was correlated with Cu, I, Mo, Mn, Ni, Sb, Cd and Sn urinary levels of these elements, decreasing with age. No sex-related differences were observed. Finally, the weight and diet group were relevant predictors for Cu, Ni, Sb and As. Significant differences were observed between the overweight/obese children on a healthy diet and the normal/underweight children on a normal diet for Cu, Ni, Sb and As. With the healthy diet/obese and overweight group presenting higher values. The present work highlights the need for further studies to assess possible sources of exposure for the Portuguese pediatric population to prevent harmful exposures.

### Strength and Limitations

The strength of this study lies in presenting for the first time a report on several urinary trace element concentrations for Portuguese children, thereby imparting preliminary insights into the exposure landscape within the scarcely investigated Portuguese population.

Some limitations of this study should be noted. The complex nature of the original study design (determination of exposure to suspected or confirmed predominantly persistent endocrine disruptor and/or obesogens) was less suitable to analyze effect modifications with urinary trace element concentrations because of its short half-life. Urine samples were not collected before the nutritional guidance started, nor was a dietary questionnaire applied, and thus, it was not possible to address the direct effect of the type of the diet to the exposure levels. Additionally, the inclusion of other trace elements, such as Hg, Zn, Se, Na, K, Ca and Fe, could have strengthened the exposure characterization and evaluation in this population. The age range of 4–18 years includes a diverse population, encompassing not only school-aged children but also infants and adolescents. This heterogeneity within the age range should be recognized as a potential limitation of the study. However, age was considered when performing the statical analysis. The sample size was not sufficient for its extrapolation to the broader Portuguese population, but it allowed preliminary insights into the exposure of these populations.

## Figures and Tables

**Table 1:** General characteristics of the studied subjects.

	Healthy Diet/Obese and Overweight (n = 67) ^a^			Regular Diet/Normal Weight (n = 43) ^a^			Total Sample (n = 110)			*p* Value *
	Median	95th	Max	Median	95th	Max	Median	95th	Max	
Age (years)	9.00	16	17.0	11.0	17	18.0	10.0	16.5	18.0	0.422
Sex (%)	Female 44% Male 56%			Female 50% Male 50%			Female 50% Male 50%			
**Weight (kg)**	45.5	82.3	120	34.8	64.8	75.0	44.3	80.8	120	≤0.001
Height (cm)	141	168	182	143	181	184	142	172	184	0.670
**BMI (kg/m^2^)**	24.6	34.4	42.3	17.1	22.9	24.1	21.9	30.5	42.3	≤0.001
Creatinine (g/L)	0.95	3.81	2.82	0.88	2.55	2.64	0.92	2.60	3.81	0.790

^a^ The obese/overweight and underweight/normal weight groups were defined according to the WHO charters [38]; * Mann–Whitney U test, 2 tailed; significant differences between the healthy and regular diet groups *(p* ≤ 0.05) are marked in bold.

**Table 4:** Varimax rotated principal component-factor loading scores.

	Factor 1	Factor 2	Factor 3	Factor 4	Factor 5
**Weight and diet group**	−0.514	0.199	**0.383**	−0.127	**0.57**
**Region**	−0.437	−0.034	**0.725**	0.051	−0.012
Age	−0.202	−0.773	0.053	−0.014	0.275
**Cu**	**0.802**	**0.35**	−0.062	−0.002	0.092
**Co**	0.221	0.047	**0.775**	−0.119	0.128
**I**	0.267	**0.683**	0.269	−0.082	0.078
**Mo**	0.155	**0.622**	0.096	**0.618**	−0.015
**Mn**	**0.76**	0.067	0.126	0.113	0.014
**Ni**	**0.605**	0.092	**0.577**	0.044	0.077
**Sb**	**0.797**	**0.404**	0.057	0.11	0.087
**As**	**0.384**	−0.127	0.078	0.093	**0.786**
**Cd**	0.246	**0.524**	0.239	**0.629**	0.101
**Pb**	**0.489**	0.198	−0.120	−0.170	0.16
Sn	0.074	0.146	0.175	−0.671	0.00
**Tl**	0.134	**0.561**	−0.322	−0.002	**0.332**

Extraction method: principal component analysis. Rotation method: varimax with Kaiser normalization. In bold, factor loading scores >0.3.

**Table 5:** Results of multiple log-linear regression models showing significant predictors (diet group, region, and age) on trace element urinary concentrations (n = 110).

Elements	Factors	Unstandardized Coefficients		Standardized Coefficients	t	Sig.	95% Confidence Interval for ß		R	Adj R	95% Confidence Interval for ß		gMR
		ß	Std. Error	ß			Lower Bound	Upper Bound			Lower Bound	Upper Bound	
Cu	Diet and weight group	−0.38	0.12	−0.31	−3.08	0.003	−0.62	0.13	0.436	0.420	0.54	1.14	0.69
	Region	−0.33	0.13	−0.25	−2.45	0.016	−0.59	−0.06			0.55	0.94	0.72
	Age	−0.06	0.01	−0.33	−4.45	<0.0001	−0.08	−0.03			0.92	0.97	0.94
Co	Diet and weight group	−0.20	0.14	−0.18	−1.40	0.164	−0.47	0.08	0.116	0.091	0.62	1.08	0.82
	Region	0.53	0.15	0.44	3.48	<0.0001	0.23	0.83			1.26	2.29	1.70
	Age	−0.01	0.02	−0.04	−0.47	0.643	−0.04	0.02			0.96	1.02	0.99
I	Diet and weight group	0.05	0.15	0.04	0.34	0.736	−0.24	0.34	0.340	0.321	0.79	1.40	1.05
	Region	0.15	0.16	0.11	0.97	0.336	−0.16	0.47			0.85	1.60	1.17
	Age	−0.12	0.02	−0.59	−7.27	<0.0001	−0.15	−0.08			0.86	0.92	0.89
Mo	Diet and weight group	−0.15	0.11	−0.16	−1.32	0.191	−0.37	0.07	0.194	0.172	0.69	1.08	0.86
	Region	0.10	0.12	0.10	0.85	0.397	−0.14	0.34			0.87	1.41	1.11
	Age	−0.06	0.01	−0.41	−4.56	<0.001	−0.08	−0.03			0.92	0.97	0.95
Mn	Diet and weight group	−0.68	0.17	−0.42	−4.04	<0.001	−1.02	−0.35	0.424	0.408	0.36	0.71	0.50
	Region	−0.10	0.19	−0.06	−0.55	0.581	−0.47	0.26			0.63	1.30	0.90
	Age	−0.09	0.02	−0.39	−5.11	<0.001	−0.13	−0.06			0.88	0.94	0.91
Ni	Diet and weight group	−0.51	0.14	−0.43	−3.54	<0.001	−0.80	−0.22	0.188	0.165	0.45	0.80	0.60
	Region	0.55	0.16	0.42	3.47	<0.001	0.23	0.86			1.26	2.35	1.72
	Age	−0.04	0.02	−0.24	−2.68	0.009	−0.07	−0.01			0.93	0.99	0.96
Sb	Diet and weight group	−0.56	0.14	−0.41	−4.11	<0.001	−0.84	−0.29	0.468	0.453	0.43	0.75	0.57
	Region	−0.02	0.15	−0.01	−0.12	0.902	−0.32	0.28			0.73	1.32	0.98
	Age	−0.10	0.02	−0.47	−6.48	<0.001	−0.13	−0.07			0.88	0.94	0.91
As	Diet and weight group	0.51	0.21	0.33	2.49	0.014	0.11	0.92	0.069	0.042	1.11	2.51	1.67
	Region	−0.57	0.23	−0.33	−2.55	0.012	−1.02	−0.13			0.36	0.88	0.56
	Age	−0.02	0.02	−0.07	−0.74	0.464	−0.06	0.03			0.94	1.03	0.98
Cd	Diet and weight group	0.03	0.10	0.03	0.27	0.788	−0.18	0.23	0.140	0.115	0.84	1.26	1.03
	Region	0.03	0.11	0.03	0.24	0.814	−0.20	0.25			0.82	1.28	1.03
	Age	−0.05	0.01	−0.38	−4.13	<0.001	−0.07	−0.02			0.94	0.98	0.96
Pb	Diet and weight group	−0.40	0.22	−0.22	−1.87	0.065	−0.83	0.03	0.218	0.196	0.44	1.03	0.67
	Region	0.00	0.24	0.00	0.01	0.994	−0.46	0.47			0.63	1.60	1.00
	Age	−0.10	0.02	−0.37	−4.20	<0.001	−0.14	−0.05			0.87	0.95	0.91
Sn	Diet and weight group	0.46	0.28	0.21	1.63	0.107	−0.10	1.02	0.104	0.079	0.90	2.76	1.58
	Region	−0.16	0.31	−0.07	−0.51	0.610	−0.77	0.45			0.46	1.57	0.85
	Age	−0.10	0.03	−0.31	−3.31	0.001	−0.16	−0.04			0.85	0.96	0.90
Tl	Diet and weight group	0.22	0.20	0.14	1.10	0.276	−0.18	0.61	0.129	0.104	0.84	1.84	1.24
	Region	−0.75	0.22	−0.43	−3.46	<0.001	−1.18	−0.32			0.31	0.73	0.47
	Age	0.02	0.02	0.10	1.09	0.276	−0.02	0.07			0.98	1.07	1.02

ß = standardized regression coefficient; beta = no standardized regression coefficient; Sig = significance; R= squared multiple correlations of predictor variable; Adj R= adjusted squared multiple correlations of predictor variable. Continuous measure of creatinine in urine children (log transformed g/L). Geographic location (Porto = 1 and Aveiro = 2). Age is categorized as 4–11 years old (=1) or 12–18 years old (=2). Gender as female (=1) and male (=2). Diet and weight group, as healthy diet group–obese/overweight children on a healthy diet (=1) and regular diet group–normal/underweight children on their regular diet (=2). Significance as *p* ≤ 0.05.

## Data Availability

Not Applicable.

## Supplementary Materials

The following supporting information can be downloaded at: https://www.mdpi.com/article/10.3390/toxics11090767/s1, Table S1: Median concentrations of trace elements (µg/g creatinine) according to diet group, region, gender, and age.
