# Supplementary material for: Trace Elements in Portuguese Children: Urinary Levels and Exposure Predictors
Source: Toxics. 2023 Sep 9;11(9):767. doi: 10.3390/toxics11090767 (PMC10535189; doi:10.3390/toxics11090767)
Supplement: Supplementary file 1 [file toxics-11-00767-s001.zip › toxics-2541422-supplementary.pdf]

Supplementary Material

Trace Elements in Portuguese Children: Urinary Levels and Exposure Predictors

Luísa Correia-Sá [1,*], Virgínia C. Fernandes [1,2], Maria Luz Maia [1,2], Edgar Pinto [3,4], Sónia Norberto [2], Agostinho Almeida [3], Cristina Santos [2,5], Cristina Delerue-Matos [1], Conceição Calhau [2,6] and Valentina F. Domingues [1,*]

1 REQUIMTE/LAQV, Instituto Superior de Engenharia do Porto, Instituto Politécnico do Porto, Rua Dr. António Bernardino de Almeida 431, 4249-015 Porto, Portugal; virginiacruz@graq.isep.ipp.pt (V.C.F.); mariadaluz.maia@gmail.com (M.L.M.); cmm@isep.ipp.pt (C.D.-M.)

2 Center for Research in Health Technologies and Information Systems, 4200-450 Porto, Portugal; sonia.norberto.nutri@gmail.com (S.N.); csantos.cristina@gmail.com (C.S.); ccalhau@nms.unl.pt (C.C.)

3 REQUIMTE/LAQV, Departamento de Ciências Químicas, Faculdade de Farmácia, Universidade do Porto, 4249-015 Porto, Portugal; ecp@ess.ipp.pt (E.P.); aalmeida@ff.up.pt (A.A.)

4 Department of Environmental Health, School of Health, P.Porto, R. Dr. António Bernardino de Almeida 400, 4200-072 Porto, Portugal

5 Health Information and Decision Science, Faculty of Medicine, University of Porto, 4200-319 Porto, Portugal

6 Nutrição e Metabolismo NOVA Medical School, Faculdade de Ciências Médicas, Universidade Nova de Lisboa, 1169-056 Lisboa, Portugal

* Correspondence: mariacs31@gmail.com (L.C.-S.); vfd@isep.ipp.pt (V.F.D.)

Keywords: elements; biomonitoring; children; obesity; predictors; urinary levels

Table S1- Median concentrations of trace elements (µg/g creatinine) according to diet group, region, gender, and age.

Element	Diet group					Region					Gender					Age				
	Healthy diet (n=67)		Regular diet (n=43)		p-value	Porto (n=79)		Aveiro (n= 31)		p-value	Female (n=55)		Male (n=55)		p-value	4-11 years		12-18 years		p-value
	Median	Max.	Median	Max.		Median	Max.	Median	Max.		Median	Max.	Median	Max.		Median	Max.	Median	Max.	
**Cu**	**27.2**	**94.4**	**14.7**	**74.2**	**<0.001**	**26.2**	**94.4**	**12.**	**35.32**	**<0.001**	22.1	94.4	21.7	62.2	0.388	**26.7**	**94.4**	**13.0**	**74.2**	**<0.001**
**Co**	0.74	1.62	0.88	2.86	0.147	**0.66**	**2.15**	**0.90**	**2.86**	**0.002**	0.83	2.86	0.74	1.90	0.233	0.84	1.89	0.65	2.86	0.214
**I**	118.6	536.9	131.9	355.6	0.126	122.5	536.9	131.9	310.3	0.311	118.6	435.2	128.7	536.9	0.832	**152.4**	**536.9**	**62.2**	**355.6**	**<0.001**
**Mo**	55.9	149.8	51.4	130.4	0.431	55.0	149.8	51.4	117.6	0.534	55.	149.8	51.7	130.4	0.950	**63.1**	**149.8**	**44.1**	**110.4**	**<0.001**
**Mn**	**2.89**	**27.9**	**1.44**	**8.75**	**<0.001**	**2.50**	**14.0**	**1.05**	**27.9**	**<0.001**	2.32	11.5	1.92	27.9	0.213	**2.91**	**27.9**	**1.19**	**8.75**	**<0.001**
**Ni**	4.83	15.4	4.65	24.8	0.238	4.65	17.0	4.78	24.8	0.401	5.14	24.8	4.47	13.8	0.174	**5.47**	**24.8**	**3.48**	**17.0**	**<0.001**
**As**	31.8	214.6	48.7	453.6	0.175	40.3	453.6	29.7	166.1	0.246	36.5	453.6	39.3	236.6	0.988	39.9	214.6	32.3	453.6	0.219
**Sb**	**0.10**	**0.32**	**0.06**	0.31	**0.003**	**0.10**	**0.32**	**0.06**	**0.31**	**<0.001**	0.09	0.32	0.08	0.31	0.435	**0.11**	**0.32**	**0.05**	**0.31**	**<0.001**
**Cd**	0.29	0.72	0.29	0.64	0.963	0.29	0.59	0.25	0.72	0.727	0.29	0.58	0.29	0.72	0.790	**0.32**	**0.72**	**0.23**	**0.47**	**<0.001**
**Pb**	**1.21**	**19.9**	**0.82**	**10.2**	**0.050**	**1.10**	**19.9**	**0.79**	**7.87**	**0.044**	0.99	19.88	0.88	6.61	0.368	**1.24**	**19.9**	**0.62**	**10.1**	**<0.001**
**Sn**	**0.39**	**30.1**	**0.49**	11.8	**0.038**	0.45	30.06	0.46	11.8	0.659	0.49	11.80	0.40	30.06	0.233	**0.49**	**30.1**	**0.25**	**11.8**	**0.003**
**Tl**	0.42	2.68	0.36	2.49	0.357	**0.46**	**2.68**	**0.22**	**0.99**	**<0.001**	0.41	2.68	0.37	1.57	0.404	**0.48**	**2.68**	**0.26**	**1.12**	**<0.001**

Mann-Whitney-U test, 2 tailed; significant differences between groups ($p \leq 0.05$) are marked in bold. Healthy diet – obese/overweight children on healthy diet; Regular diet – normal/underweight children following their regular diet; Max. - Maximum
